# Systematic study of TiO_2_/ZnO mixed metal oxides for CO_2_ photoreduction†

**DOI:** 10.1039/c9ra03435h

**Published:** 2019-07-12

**Authors:** Warren A. Thompson, Alberto Olivo, Danny Zanardo, Giuseppe Cruciani, Federica Menegazzo, Michela Signoretto, M. Mercedes Maroto-Valer

**Affiliations:** Research Centre for Carbon Solutions (RCCS), School of Engineering & Physical Sciences, HeriotWatt University Edinburgh EH14 4AS UK wat1@hw.ac.uk; Low Emission Resources Corporation 2 Mid Craigie Rd Dundee DD4 7RH UK; CatMat Lab, Dept. of Molecular Sciences and Nanosystems, Ca' Foscari University Venice, Consortium INSTM, RU of Venice Via Torino 155 30172 Venezia Italy; Department of Physics and Earth Sciences, University of Ferrara Via G. Saragat 1 Ferrara I-44122 Italy

## Abstract

A two component three degree simplex lattice experimental design was employed to evaluate the impact of different mixing fractions of TiO_2_ and ZnO on an ordered mesoporous SBA-15 support for CO_2_ photoreduction. It was anticipated that the combined advantages of TiO_2_ and ZnO: low cost, non-toxicity and combined electronic properties would facilitate CO_2_ photoreduction. The fraction of ZnO had a statistically dominant impact on maximum CO_2_ adsorption (*β*_2_ = 22.65, *p*-value = 1.39 × 10^−4^). The fraction of TiO_2_ used had a statistically significant positive impact on CO (*β*_1_ = 9.71, *p*-value = 2.93 × 10^−4^) and CH_4_ (*β*_1_ = 1.43, *p*-value = 1.35 × 10^−3^) cumulative production. A negative impact, from the interaction term between the fractions of TiO_2_ and ZnO, was found for CH_4_ cumulative production (*β*_3_ = −2.64, *p*-value = 2.30 × 10^−2^). The systematic study provided evidence for the possible loss in CO_2_ photoreduction activity from sulphate groups introduced during the synthesis of ZnO. The decrease in activity is attributed to the presence of sulphate species in the ZnO prepared, which may possibly act as charge carrier and/or radical intermediate scavengers.

## Abstract 2

A novel example using a systematic design of experiments mixture design for developing mixed metal oxide photocatalysts for CO_2_ photoreduction.

## Graphical abstract

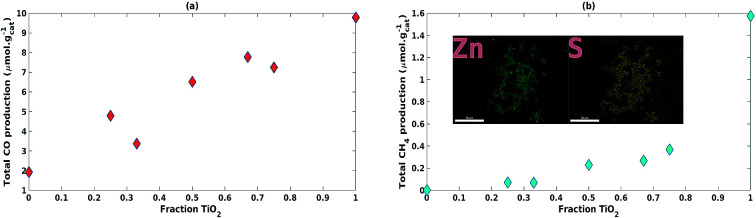

## 1 Introduction

CO_2_ photoreduction is one of the potential technologies for carbon utilisation.^1^ However, major optimization in photocatalyst design is required for its applicability.^2^ Possible approaches in heterogeneous photocatalysis to improve photocatalytic activity include photocatalyst dispersion on highly porous substrates and the use of coupling two semiconductors as photocatalysts. For these reasons, composite mixtures of ZnO and TiO_2_ were prepared on an ordered mesoporous SBA-15 silica support for CO_2_ photoreduction. SBA-15 was chosen as it has several favourable characteristics including: a large surface area which may enhance photocatalyst dispersion and the availability of photons;^3^ SBA-15 is also chemically and mechanically stable^4^ and SBA-15 has shown effectiveness as a CO_2_ photoreduction support.^5–7^

TiO_2_ has been shown to be an effective photocatalyst for CO_2_ photoreduction with numerous examples found in the literature.^1,8,9^ ZnO has also shown promise as a photocatalyst for CO_2_ photoreduction.^10–12^ ZnO offers improved CO_2_ adsorption^12^ and low charge carrier recombination.^13^ Both TiO_2_ and ZnO share low cost, non-toxicity and relatively environmentally friendly properties.^1,14^

TiO_2_ is not efficient for CO_2_ photoreduction due to: poor charge carrier mobility leading to a fast recombination rate^13^ and hindered CO_2_ adsorption in the presence of H_2_O due to the limited presence of surface basic functionalities.^15^ On the contrary, ZnO exhibits a longer charge carrier lifetime^16^ and suitable surface basicity,^17^ which can improve CO_2_ adsorption. Moreover, the coupling of TiO_2_ and ZnO, was reported to form a heterojunction that could reduce charge carrier recombination leading to enhanced CO_2_ photoreduction activity.^18^ Composite mixtures of anatase TiO_2_ and wurtzite-type ZnO, due to the TiO_2_/ZnO heterojunction formed, showed improved CO_2_ photoreduction activity.^19^ Other examples of composite mixtures of TiO_2_ and ZnO leading to improved photocatalytic activity, due to less charge recombination, include the degradation of phenols and salicylic acid.^20,21^ Due to their synergistic effects on electronic and acid/base properties, the use of TiO_2_ and ZnO as photocatalyst mixture is promising for CO_2_ photoreduction. However, no examples have described the impact of different mixing fractions of TiO_2_ and ZnO on CO_2_ photoreduction performance.

CO_2_ photoreduction faces the challenge of low efficiency but also the deactivation of the photocatalyst.^22^ Deactivation of the photocatalyst has been reported, especially when production data is collected in continuous flow setups, for CO_2_ photoreduction by a growing number of authors.^23–32^ Possible explanations for deactivation include: photocatalyst poisoning due to irreversible adsorption of reaction intermediates; sintering and agglomeration of the photocatalyst metal active sites and loss of active reaction sites that include oxygen vacancies, surface hydroxyls and Ti^3+^ sites.^22^ To develop CO_2_ photoreduction, low efficiency and deactivation of the photocatalyst need to be addressed. In a closely related field of photocatalytic oxidation, radical scavenging of the reactive oxygen species as hydroxyl radicals have been found to lead to deactivation.^33^ Some inorganic anions are known to interact with radical processes, by yielding less reactive and more stable intermediates and thus hampering the overall photocatalytic reaction.^34^

High throughout technologies and automation are critical to finding suitable photocatalysts.^35^ Central to these technologies is the use of systematic experimental designs, Design of Experiments (DOE), for decision making. There are numerous examples in the literature describing the use of DOE for engineering and process optimisation.^29,36,37^ Mixture designs can efficiently evaluate the impact of component fractions in a mixture.^38^ In this work, the impact of TiO_2_ and ZnO fractions used for the formulation of a mixed metal oxide (MO) photocatalyst mixture on a SBA-15 support, was evaluated for CO_2_ photoreduction using a novel combination of a systematic mixture design and photocatalysis theory.

In this work in-house synthesis of the photocatalysts was used due to the potential and scope, using different synthetic methodologies, for improvements to increase surface area, crystallinity,^39^ photocatalyst coverage and optical properties.^29^

## 2 Experimental

### 2.1 Photocatalyst preparation

SBA-15 was synthesized according the procedure reported in literature.^40^ Briefly, template EO20-PO70-EO20 (P123, Aldrich) was dissolved in aqueous HCl solution and tetraorthosilicate (TEOS) was introduced as silica precursors. Powder was aged at 90 °C, dried and then calcined at 550 °C for 6 h under air flow. TiO_2_ and ZnO were synthesised by precipitation of inorganic salts. In the case of TiO_2_, a titanyl sulphate solution and a NaOH solution were added dropwise to deionised H_2_O under vigorous stirring, keeping pH neutral. Then the Ti(OH)_4_ suspension was aged at 60 °C for 20 h and then washed with distilled H_2_O to remove the sulphate ions and dried at 110 °C for 18 h and finally calcined at 400 °C for 4 h in air flow.^41^ ZnO was prepared following the same procedure reported for TiO_2_, but starting from a ZnSO_4_ solution as precursor and keeping the pH slightly alkaline (pH 9) during the precipitation. The prepared TiO_2_ and ZnO were added onto SBA-15 by incipient wetness impregnation using isopropanol as a liquid medium. Samples were then dried at 110 °C for 18 h.

### 2.2 UV-vis absorption

The light absorption and electronic band were characterized using a UV-vis spectrometer (PerkinElmer lamda 950) equipped with a 150 mm integration sphere (PerkinElmer). The band gap was determined using the Kubelka–Munk function (1) and intersection of the Tauc segment and *hν*-axis of the Tauc plot.^42^1
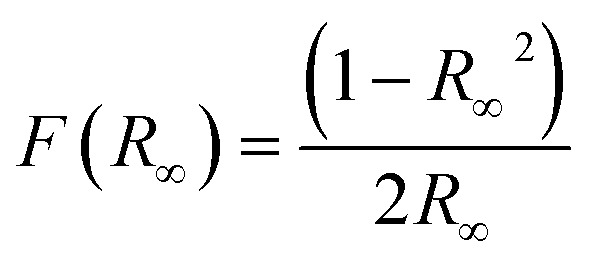
where *F*(*R*_∞_) is the reemission function and *R*_∞_ is the reflectance of the sample with infinite thickness.

### 2.3 XRD characterisation

For the analysis of mixed MOs on SBA-15, a Bruker D8 Advance powder diffractometer, operating with Ge-monochromated Cu Kα radiation (wavelength = 1.5406 Å) and a LynxEye linear detector. Data were collected over the angular range 5–85° in 2*θ*. For the analysis of pure ZnO and TiO_2_, X-ray Diffraction (XRD) patterns were collected on a Bruker D8 Advance powder diffractometer with a sealed X-ray tube (copper anode, 40 kV and 40 mA) and a Si(Li) solid state detector (Sol-X) set to discriminate the Cu Kα radiation. Apertures of divergence, receiving, and detector slits were 2.0 mm, 2.0 mm, and 0.2 mm, respectively. Data scans were performed in the 2*θ* range 5–75° with 0.02° step size and counting times of 3 s per step. Quantitative phase analysis determination performed using the Rietveld method as implemented in the TOPAS v.4 program (Bruker AXS) using the fundamental parameters approach for line-profile fitting.

### 2.4 N_2_ physisorption

Specific surface areas (SSA) of the samples were evaluated by N_2_ physisorption. 200 mg of the sample was placed under vacuum at 200 °C for 2 h. The analyses were then carried out recording the adsorption–desorption isotherm at −196 °C with a Micromeritics ASAP 2000 analyzer. SSAs were finally determined by the BET equation.^43^

### 2.5 CO_2_ adsorption

Samples were degassed under a constant purge of N_2_ at 200 °C for 10 h. CO_2_ adsorption capacities were estimated by the maximum value found from the CO_2_ adsorption isotherm measured at 273 K over fifteen equidistant points from 0 to 0.95 *P*/*P*_0_ (Gemini VII 2390).

### 2.6 CO_2_ photoreduction tests

**Fig. 1:**
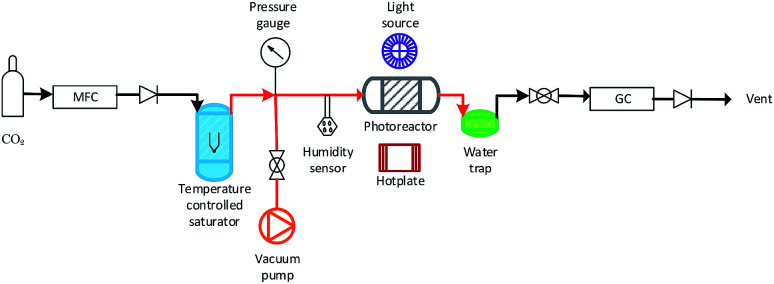
Overview of the experimental setup used for the MO photocatalyst mixture CO_2_ photoreduction tests (not to scale). Pipe lines in red were heated with a temperature controlled heating rope.

A slurry of the prepared mixed MO photocatalyst was prepared by adding ≈100 mg of the mixed MO photocatalyst to 1 ml DI H_2_O in a 5 ml vial. The vial was sealed and agitated in a ultrasonic bath for two minutes. The slurry was then deposited dropwise onto a glass fiber disc (47 mm diameter). The coated glass fiber disc was dried at 120 °C for 2 h. The coated glass fiber disc was placed in the middle of a stainless steel photoreactor (*r* = 25 mm, *h* = 1 mm, *ν* = 1.96 mm^3^) and sealed. Residual air in the system was evacuated *via* three repetitive steps of placing the system under vacuum to −1 bar and the vacuum released with CO_2_ (99.995%) to +1 bar. The flow rate of CO_2_ was set to 0.35 ml min^−1^ and passed through the temperature controlled (±0.1 °C) aluminium body saturator for at least 12 h to allow the system to equilibrate. Relative humidity (±1.8% RH) was measured using an inline Sensirion SHT75 humidity sensor potted (MG Chemicals 832HD) into a Swagelok 1/4′′ T-piece. The temperature of the photocatalyst surface (40 °C ± 2.0 °C) was controlled using a hotplate and the surface temperature measured using a Radley's pyrometer. To prevent condensation at higher saturation temperatures, the lines from the outlet of the saturator up until the inlet of the H_2_O trap were heated and temperature controlled (±0.1 °C) with a heating rope and thermocouple (Fig. 1).

An OmniCure S2000 fitted with a 365 nm filter was used as the light source and the irradiance (295.71 ± 1.60 mW cm^−2^) checked before each experiment using an OmniCure R2000 radiometer (±5%). An inline GC (Agilent, Model 7890B series) with a Hayesep Q column (1.5 m), (1/16 inch od, 1 mm id), MolSieve 13× (1.2 m), (1/16 inch od, 1 mm id), thermal conductivity detector (TCD), nickel catalysed methanizer and flame ionization detector (FID) was used to analyze the output of the photoreactor every four minutes. CO and CH_4_ production rates were recorded in units of μmol g_cat_^−1^ h^−1^ using only the mass of active mixed MO photocatalyst/s used with the exclusion of the SBA-15 support mass. Cumulative production (μmol g_cat_^−1^) was calculated by integrating the area under the production rate (μmol g_cat_^−1^ h^−1^) *vs.* time (h) curve.

### 2.7 Ionic chromatography method for testing sulphates

Quantitative analysis of sulphates was performed through a procedure previously reported for sulphate-doped zirconia.^44^ 200 mg of the sample was treated with 250 mL of 0.1 M NaOH solution to extract the sulphates. The suspension was filtered and analyzed. A LC20 ionic chromatographer equipped with a 25 μL injection loop, a AS14 separation column, a AG14 guard column, an acid resin suppressor and a ED40 conductivity detector was used. A buffer solution of 10 mM Na_2_CO_3_ and 3.5 mM NaHCO_3_ in Milli-Q H_2_O, at room temperature was used as eluent. A calibration curve for quantitative analysis was obtained using standard Na_2_SO_4_ solution between 1 and 8 ppm.

### 2.8 SEM/EDX analysis

Scanning electron microscopy and X-ray energy dispersive spectroscopy analysis of the mixed MOs and SBA support were performed using a FEI Scios SEM equipped with an EDAX Octane Plus EDS detector.

### 2.9 Design of experiments

A two component three degree simplex lattice design was employed with experimental settings and results shown in Table 1. MATLAB was used to estimate: the fitted coefficient values; determine the *p*-values and plot the models and data.

**Table 1:** Two component three degree simplex lattice design points used for experimental settings (*X*_1_ and *X*_2_) as mass fractions of TiO_2_ and ZnO respectively. Amounts of TiO_2_ and ZnO mixed with 800.0 mg SBA-15

Exp. name	*X*_1_ fraction TiO_2_	*X*_2_ fraction ZnO	Amount TiO_2_ (mg)	Amount ZnO (mg)
MO1	1.00	0.00	200.2	0.0
MO2	0.67	0.33	133.9	67.4
MO3	0.33	0.67	66.5	133.2
MO4	0.00	1.00	0.0	200.4
MO5	0.50	0.50	100.4	102.7
MO6	0.75	0.25	149.5	53.5
MO7	0.25	0.75	50.7	150.7

The experimental design results were used to fit the polynomial function shown by (2).

2 *Y* = *β*_1_*X*_1_ + *β*_2_*X*_2_ + *β*_3_*X*_1_*X*_2_

where *Y* is the cumulative production of CO or CH_4_; *X*_1_ and *X*_2_ are the fractions of TiO_2_ and ZnO respectively; *β*_1_ and *β*_2_ are the coefficients estimated for the impact of the fractions of TiO_2_ and ZnO used respectively and *β*_3_ is the coefficient estimated for the interaction term between the fraction of TiO_2_ and ZnO.

Using the matrix of *X*_1_ and *X*_2_ fractions of TiO_2_ and ZnO values shown in Table 1 and either the maximum CO_2_ adsorption, cumulative production of CO or CH_4_ production as a response shown by *Y* in (2), the coefficients *β*_1_, *β*_2_ and *β*_3_ from (2), were estimated by linear regression using a QR decomposition algorithm (*fitlm* function) in MATLAB (Table 3). The *p*-values for each coefficient were determined using the MATLAB *fitlm* function call. Using 95% confidence, *p*-values less than 0.05 indicated that the coefficient value was not equal to zero and it's associated parameter (*X*_1_, *X*_2_ or *X*_3_) had a statistically significant impact on either maximum CO_2_ adsorption, CO or CH_4_ cumulative production.

## 3 Results and discussion

### 3.1 Characterization and properties of mixed metal oxides

**Fig. 2:**
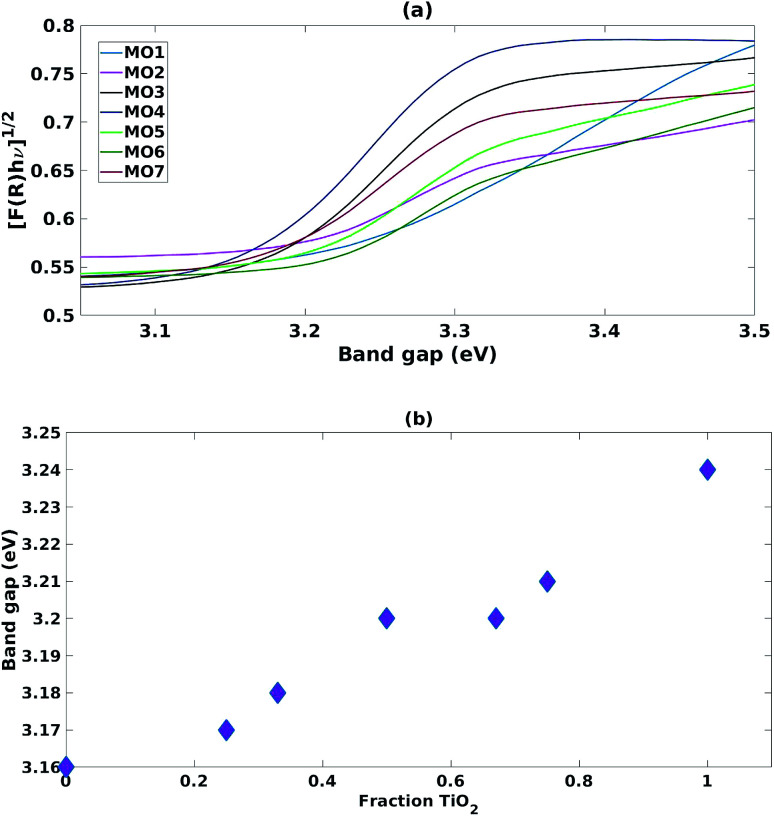
(a) Tauc plots for mixed MO photocatalysts (b) impact of increasing fraction of TiO_2_ on band gap.

The samples prepared with a high fraction of TiO_2_ (MO1, MO2, MO5 and MO6) showed the characteristic broad adsorption peak of anatase TiO_2_ (Fig. 2a). As the fraction of ZnO increased (MO3, MO4 and MO7) the Tauc plot peak shapes became sharper and characteristic of the adsorption peaks of ZnO (Fig. 2a). Increasing the fraction of TiO_2_ increased the band gap linearly from the ZnO region (3.16 eV) towards the anatase region (3.24 eV) (Fig. 2b).

**Fig. 3:**
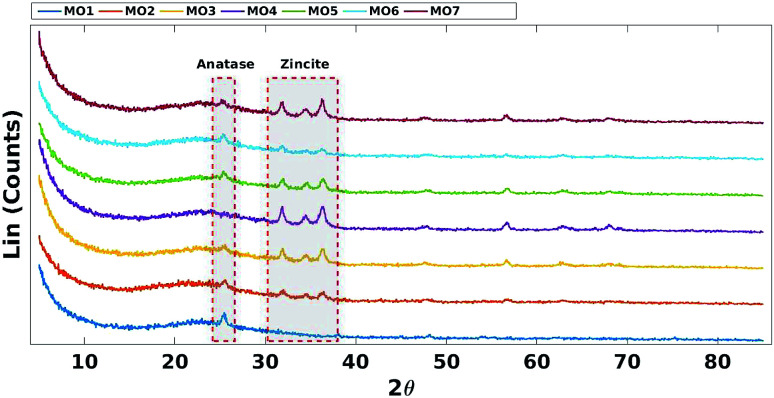
XRD comparison of mixed MO photocatalysts on SBA-15 support.

Decreasing the fraction of TiO_2_ reduced the intensity of the characteristic anatase XRD peak (JCPDS Card no. 21-1272) at 2*θ* = 25.4 (Fig. 3).^45^ Increasing the fraction of ZnO increased the intensity of the characteristic zincite peaks (JCPDS card no. 36-1451) at 2*θ* = 31.9, 34.4 and 36.2 (Fig. 3).^46^

**Fig. 4:**
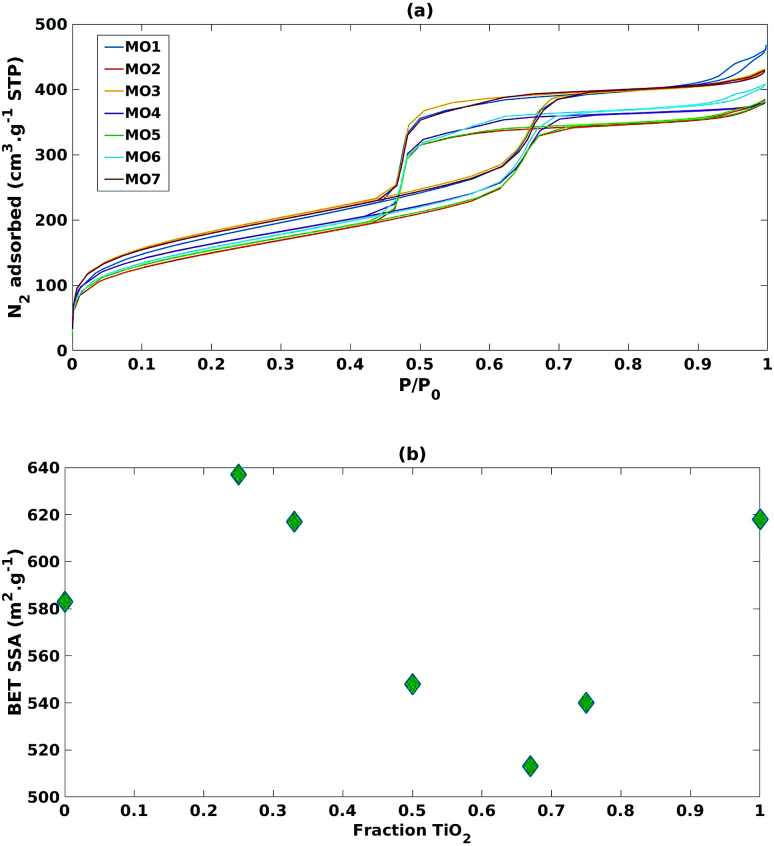
(a) N_2_ adsorption isotherms of the mixed MO photocatalysts (b) impact of increasing fraction of TiO_2_ on BET specific surface area.

As reported in Fig. 4a, adsorption isotherms of all the mixed MO samples exhibited the typical shape of SBA-15, suggesting that its ordered mesoporous structure was retained.^40^ Nevertheless, when comparing the SSAs with the TiO_2_ fraction (Fig. 4b), a sinusoidal trend was observed, suggesting that SSA has no or little effect on photoreduction efficiency and selectivity in these mixed MO systems.

### 3.2 Mixture design and the impact of TiO_2_ and ZnO fractions

#### 3.2.1 Impact TiO_2_ and ZnO fractions on CO_2_ adsorption

**Fig. 5:**
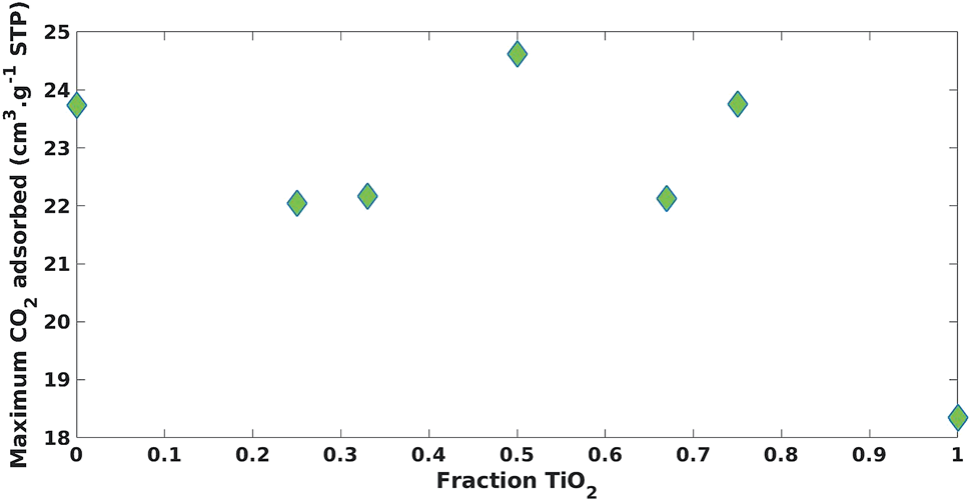
Impact of increasing fraction of TiO_2_ on of maximum CO_2_ adsorption.

Fig. 5 shows the impact of increasing the fraction of TiO_2_ used in the mixture on maximum CO_2_ adsorption. CO_2_ adsorption increased significantly when a small fraction of ZnO was present with little change with increasing the fraction of ZnO thereafter (Fig. 5).

Both the fraction of ZnO and TiO_2_ positively impacted (*β*_1_ = 19.31, *β*_2_ = 22.65) maximum CO_2_ adsorption with statistical significance (*p*-value = 2.61 × 10^−4^, *p*-value = 1.39 × 10^−4^), respectively (Table 2). The impact of the ZnO fraction had a larger coefficient value (*β*_2_ = 22.65) *versus* TiO_2_ (*β*_1_ = 19.31) and this could be explained by the increase in surface basicity.^17^ It was expected that an increase in CO_2_ adsorption would increase CO_2_ photoreduction photocatalytic activity. However, photocatalytic processes are complicated and often multiple properties of the photocatalyst need to be considered.^1^

**Table 2:** Coefficient values estimated for fitting model (2) and their respective *p*-values (*p-value < 0.05) on maximum CO_2_ adsorption

Regression results for maximum CO_2_ adsorption		
Parameter coefficient	Value estimated	*p*-Value
*β*_1_	19.31	2.61 × 10^−4^*
*β*_2_	22.65	1.39 × 10^−4^*
*β*_3_	9.28	2.32 × 10^−1^

#### 3.2.2 Impact TiO_2_ and ZnO fractions on CO_2_ photoreduction

**Fig. 6:**
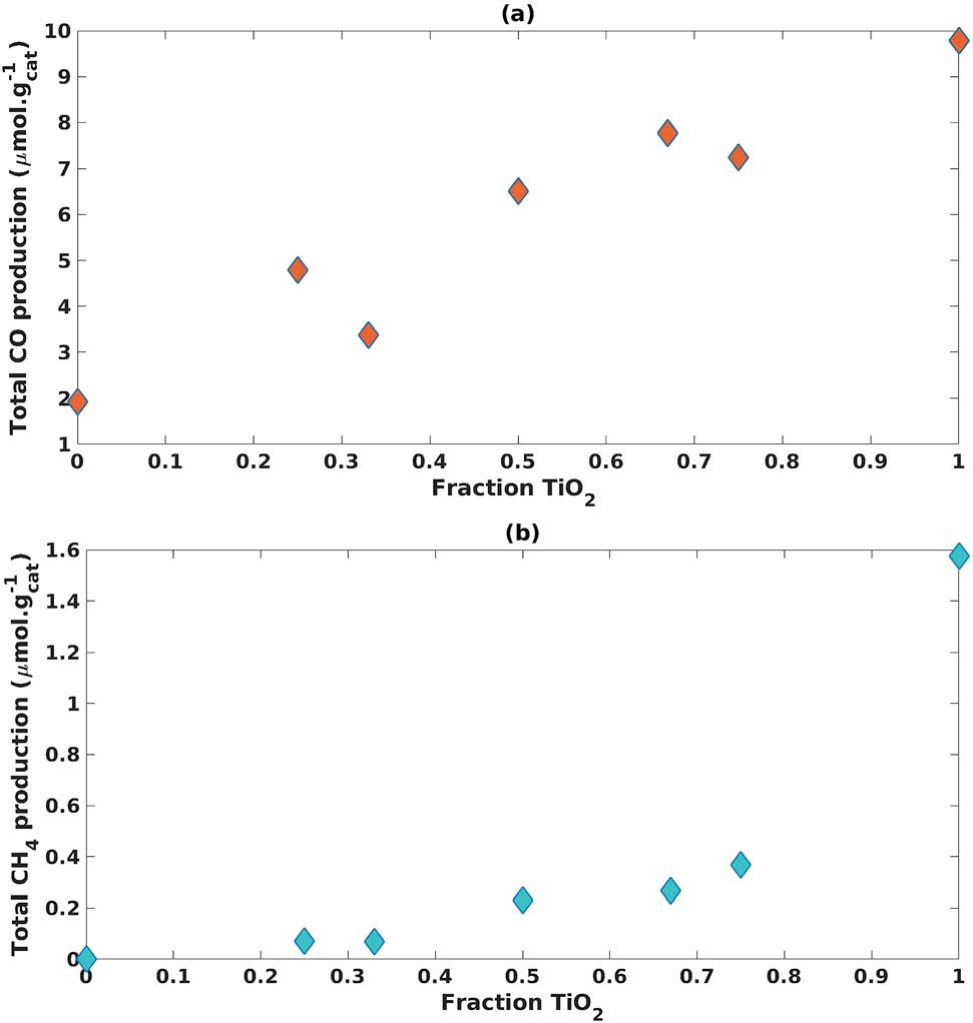
Impact of increasing fraction of TiO_2_ on (a) CO cumulative production and (b) CH_4_ cumulative production.

Fig. 6a and b shows the impact of increasing the fraction of TiO_2_ used in the mixture on CO and CH_4_ production, respectively. Increasing the fraction of TiO_2_ increased CO cumulative production with a slight curvature that closely resembled a linear trend (Fig. 6a). Eliminating ZnO from the photocatalyst mixture yielded a significant increase in CH_4_ cumulative production with a trend resembling an exponential curve (Fig. 6b).

The TiO_2_ fraction in the photocatalyst mixture positively impacted (*β*_1_ = 9.71) CO cumulative production with statistical significance (*p*-value = 2.93 × 10^−4^) (Table 3). This was also the case for CH_4_ cumulative production (*β*_1_ = 1.43, *p*-value = 1.35 × 10^−3^) (Table 3).

**Table 3:** Coefficient values estimated for fitting model (2) and their respective *p*-values (*p-value < 0.05) on CO and CH_4_ cumulative production

Regression results for CO cumulative production			Regression results for CH_4_ cumulative production		
Parameter coefficient	Value estimated	*p*-Value	Parameter coefficient	Value estimated	*p*-Value
*β*_1_	9.71	2.93 × 10^−4^*	*β*_1_	1.43	1.35 × 10^−3^*
*β*_2_	1.96	7.51 × 10^−2^	*β*_2_	0.12	5.50 × 10^−1^
*β*_3_	0.53	8.83 × 10^−1^	*β*_3_	−2.64	2.30 × 10^−2^*

An interaction effect was found between the fractions of TiO_2_ and ZnO used in the photocatalyst mixture with a statistically significant (*p*-value = 2.30 × 10^−2^) and negative impact (*β*_3_ = −2.64) on CH_4_ cumulative production (Table 3). This would indicate that the inclusion of ZnO significantly hampered the production of CH_4_.

**Fig. 7:**
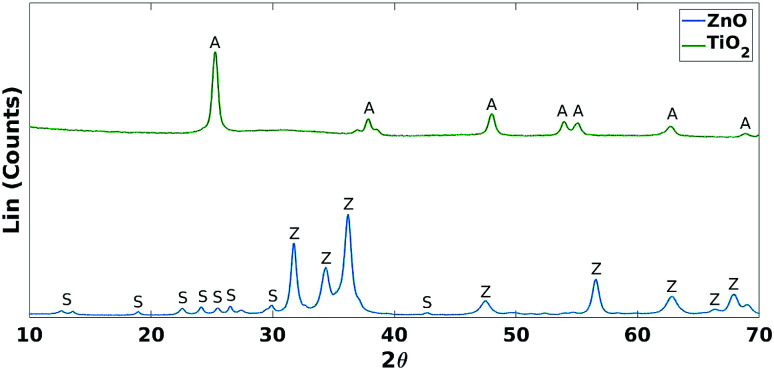
XRD comparison of TiO_2_ and ZnO. A = anatase (TiO_2_ phase), Z = zincite (ZnO phase) and S = Zn_3_O(SO_4_)_2_.

**Fig. 8:**
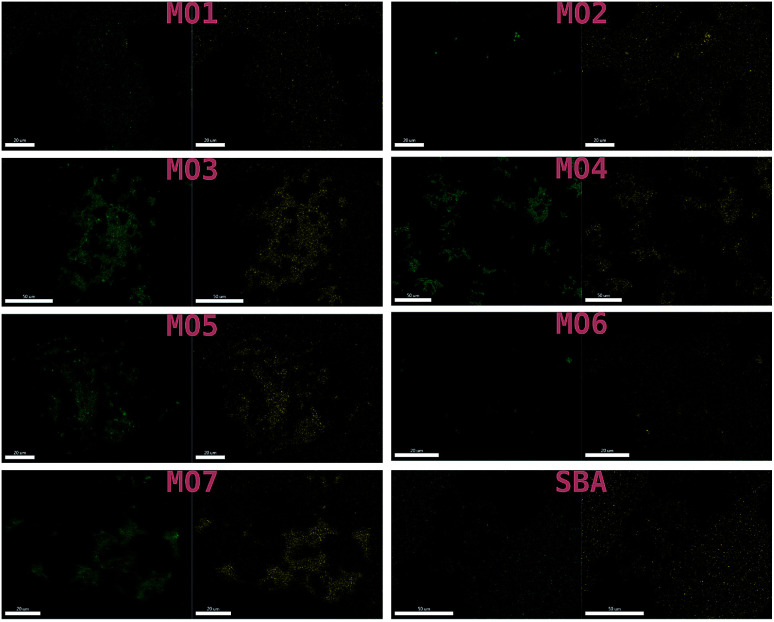
SEM/EDX of MO1–MO7 and the SBA-15 support used. Zinc mapped on the left and sulphur on the right.

**Fig. 9:**
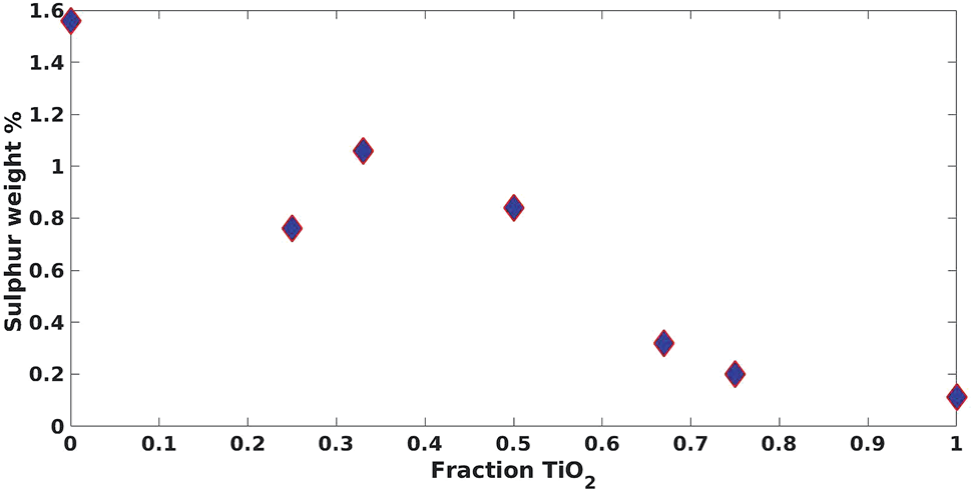
Impact of increasing fraction of TiO_2_ on approximated sulphur weight% from SEM/EDX.

These results were not encouraging from an activity point of view but they offered an opportunity for further scientific enquiry. Both TiO_2_ and ZnO were synthesised using a precipitation method that employed sulphate salts TiOSO_4_ and ZnSO_4_, respectively. Ion chromatography (IC) analyses were performed on pristine TiO_2_ and ZnO samples, showing 0.4% and 12.0% wt sulphates, respectively. The large amount of sulphates observed in the ZnO samples, was also confirmed by XRD analysis (Fig. 7), showing that this material is actually composed of 43% Zn_3_O(SO_4_)_2_, corresponding to 20.7% wt amount of sulphates, and 57% ZnO.^47^ The difference in the amount of sulphates recorded by IC and XRD is likely due to the inability of the IC analysis extraction procedure to recover all the sulphates. Sulphur and zinc mapped very closely to one another by SEM/EDX analysis (Fig. 8). Visually, sulphur content increased with increasing fraction of ZnO (Fig. 8). The EDX analysis also yielded a linear increase in sulphur with increasing the fraction of ZnO used (Fig. 9). Together, these were additional pieces of evidence highlighting the incorporation of sulphates by the ZnO used.

**Fig. 10:**
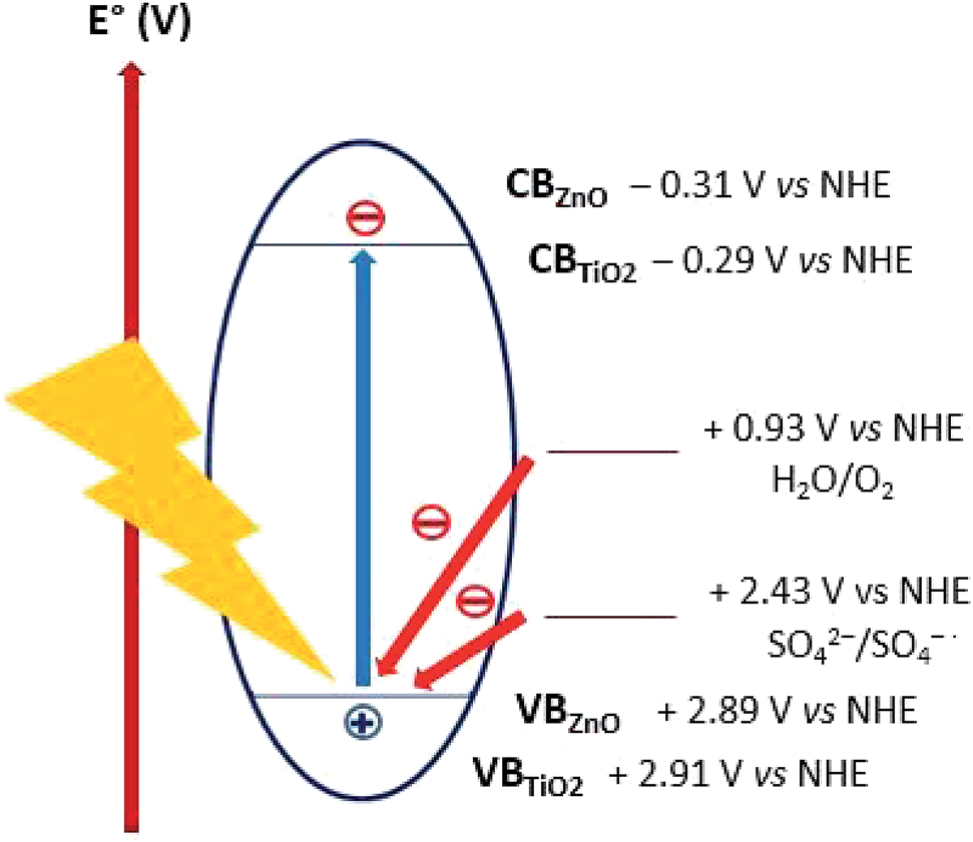
Energy levels scheme for the proposed mechanism of sulphates as hole scavengers.

Lo *et al.* reported acidic sulphate modified titania as an efficient photocatalyst for CO_2_ photoreduction^48^ Nevertheless, sulphate anions was observed to have a detrimental effect on photooxidation by acting as both radical scavenger^49^ and competing with reagents for adsorption to active photocatalyst sites.^50^ We can discount the latter hypothesis since as discussed in Section 3.2.1, ZnO was observed to improved CO_2_ adsorption. The radical (or hole) scavenging hypothesis was thus considered. Several mechanisms, all involving radical intermediates, have been proposed for CO_2_ photoreduction.^22,51^ Sulphates or species arising from radical scavenging yielding SO_4_˙^−^ species might interfere with the CO_2_ photoreduction reaction pathway. Moreover, the oxidizing holes generated on both TiO_2_ (+2.91 V *vs.* NHE) and ZnO (+2.89 V *vs.* NHE) valence band,^18^ can be potentially scavenged by sulphates (*E*° = +2.43 V *vs.* NHE),^52^ thus acting as charge carrier trap and competing with water oxidation (Fig. 10). The sulphates acting as radical and/or hole scavengers are very likely to undergo chemical transformations towards reduced sulphur species such as H_2_S, SO_2_ and S. To confirm this hypothesis, future work would include attempting to identify these species formed during the CO_2_ photoreduction reaction.

## 4 Conclusion

A systematic experimental mixture design as used to investigate the impact of the fractions of TiO_2_ and ZnO as mixed MOs on an ordered SBA-15 mesoporous support for CO_2_ photoreduction activity. The combination of a systematic experimental mixture design using numerical tools and the analysis of the prepared TiO_2_/ZnO photocatalyst properties offered an opportunity to provide evidence for the trapping of radical CO_2_ photoreduction intermediates and/or charge carriers by sulphate groups. This approach has shown use for rapid screening and the development of mixed MOs for CO_2_ photoreduction.

Increasing the fraction of ZnO increased the adsorption of CO_2_ with statistical confirmation using the mixture design. Increasing the fraction of TiO_2_ improved the production of CO with a linear trend observed. Increasing the fraction of TiO_2_ also improved the production of CH_4_ with an exponential trend observed. This was confirmed by numerical analysis where the fraction of TiO_2_ was found to be statistically significant for both CO and CH_4_ cumulative production. The exponential trend for CH_4_ cumulative production could be explained by the statistical significance of a negative interaction between the fraction of TiO_2_ and ZnO used. Increasing the fraction of ZnO yielded significantly less CH_4_ production and had a slightly less dramatic, albeit still negative, impact on CO production.

The impact of radical scavengers on deactivation has not been explored for CO_2_ photoreduction. The mixed MO mixtures was initially intended to improve the efficiency of CO_2_ photoreduction. However, this study showed how the inclusion of sulphates from the synthesis method very likely led to deactivation and lower production of CH_4_ and CO. In addition, this study serves as a framework for the efficient and systematic study of other novel photocatalyst synthetic techniques and subsequent formulation of novel mixtures for CO_2_ photoreduction.

## Conflicts of interest

There are no conflicts to declare.

## Supplementary Material

RA-009-C9RA03435H-s001
